# HIV/AIDS Awareness Among Young Adults in Hong Kong: The Roles of Knowledge, Acceptance and Stigma

**DOI:** 10.3390/ijerph18147473

**Published:** 2021-07-13

**Authors:** Fan Zhang, Louisa Chung

**Affiliations:** 1 Department of Public Health and Preventive Medicine, School of Medicine, Jinan University, Guangzhou 510632, China; fanzhang@jnu.edu.cn; 2 The Department of Health and Physical Education, The Education University of Hong Kong, Taipo, Hong Kong

**Keywords:** HIV, knowledge, acceptance, stigma, awareness, college students

## Abstract

In the past decade, HIV (Human Immunodeficiency Virus) infection risk and the prevalence of infected cases in the younger generation have increased in Hong Kong. To promote HIV prevention and control, it is critical to understand the situation of HIV-related knowledge, stigma, and awareness among the younger adults, especially college students. 810 college students (mean age = 20.63 ± 2.05) have participated in the current survey. In mediation pathway analysis, the results show that participants’ greater knowledge about HIV is associated with higher awareness, and this relationship is partially mediated by the knowledge-related increase in accepting attitudes toward the people with HIV, but not via reducing stigma. Our findings provide an updated profile of HIV-related knowledge, attitude and awareness among college students in Hong Kong. In addition, we have clarified the mediating role of acceptance in the relationship between knowledge and awareness and pinpointed the importance of knowledge education and workshops to promote acceptance of people with HIV. Insights were provided for tailoring health-promotion programs to reduce risky sex and prevent HIV infection on college campuses.

## 1. Introduction

Young people in China are now facing a rising risk of human immunodeficiency virus infection (HIV, [1]). The number of new HIV cases in people aged 15 to 24 has doubled from 8300 in 2008 to 17,000 in 2015 [2,3]. Among this population, college and university students have become particularly vulnerable, mainly due to liberal attitudes toward sex, lack of knowledge about the risk of infection [4], and the resultant higher rate of participating in unsafe sexual practice (especially under the influence of alcohol or peer pressures [5]). In Hong Kong, the prevalence of HIV is relatively low, and 10,280 cases of HIV infection have been reported cumulatively from 1984 to December 2019 according to Hong Kong HIV quarterly update. However, it has been noted that among the reported cases, the proportion of the age group of 20–29 years has increased significantly in the past decade, becoming one of the largest groups of HIV infected population [6]. To better prevent and control infection among young people in Hong Kong, especially college students, it is pivotal to understand the factors influencing their awareness of HIV, in particular, their knowledge and attitudes about HIV.

HIV-related awareness in the current study refers to the perceptions about the epidemic of HIV and the public policy (e.g., “strengthen the publicity of AIDS”, or “The government should provide more resources to people living with HIV”) toward the population living with the infection. Recent research shows that the awareness, especially the awareness of transmission risk, could modify the effects of knowledge on young people’s risky sex [7]. In particular, higher level of HIV knowledge is associated with less engagement in risky sex among female college students only when the risk perception is high [7]. Meanwhile, low awareness per se could also contribute to young people’s higher engagement in risky sexual behaviors, which is potentially related to the outbreak of HIV infection [8]. Among the reported cases of HIV infection in Hong Kong, over 78% acquired the infection via unprotected sex [6]. One of the causes for this is that the attitude toward sex has become more liberal among the young generations, while the awareness of infection risk of HIV or other sexually transmitted diseases (STD) has remained limited [9,10]. According to the Hong Kong Youth Sexuality Survey among 1127 unmarried youth (aged from 18 to 27), over 60% accepted premarital sex, and approximately 20% showed a liberal attitude toward taking sexual risks (e.g., having multiple sex partners, [11]). The changes in attitudes have inherently increased the exposure to risk-taking scenarios in sex [12].

The majority of existing literature on college students’ attitude and knowledge about HIV has focused on the descriptive analysis of the issue [12,13], and it has still been unclear about how the two constructs interact in forming young people’s awareness. In addition, with limited sex education at school or from parents [14], college students usually lack sufficient knowledge about safe sex and the HIV transmission routes [15]. The resultant misunderstandings about HIV could lead to higher propensity to isolate people with HIV infections, suggesting a stronger stigmatizing attitude [16]. A previous study has found that stigma and misunderstandings about people with HIV are widely held by college students in Chinese societies [17]. Moreover, the misconceptions about transmission routes of HIV were a common cause for greater stigma [18]. A variety of negative effects has been reported of stigma toward HIV [19]. On the one hand, stigma is likely to undermine the physical and mental health of people living with HIV (PLWH). This could also result in reduced social support and social isolation for PLWH, which may decrease their medication adherences [20], as well as their tendency to approach or retain HIV care [21,22]. On the other hand, social stigma could also lead to psychological distress and depressive symptoms among the PLWH, which further contributes to greater engagement in risky sexual behaviors [23], and potentially affecting more people. Meanwhile, stigma often has a negative association with HIV awareness [24], though the causal effect was not clear. 

The existing literature on HIV-related knowledge, attitudes, and awareness among Hong Kong college students has been relatively sparse and outdated, compared with the vast number of studies in regions with higher prevalence of HIV infection, e.g., Africa [25]. To fill this research gap, the present study aimed to assess college students’ level of knowledge about and attitudes toward HIV, as well as to investigate how the two factors may interplay in influencing their awareness about the epidemic influences and public policy of HIV. The findings will provide critical insights for developing health promotion programs to prevent and control the spread of HIV among the young generation in Hong Kong. 

## 2. Materials and Methods

### 2.1. Design and Participants

A cross-sectional survey using structured questionnaires was conducted among college students from eight universities in Hong Kong. With a random sampling, college students were recruited at a balanced ratio between male and female. The eligible participants were (a) 18 years or above and (b) could speak fluent Chinese. A written consent form was obtained from the participants before taking part in the study, and no compensation was provided for this. The project has been approved by the Ethics of Honor Project at departmental level, the Education University of Hong Kong. 

### 2.2. Measures

Demographic information included age; gender (male = 1, female = 2, transgender = 3); education (lower than bachelor’s degree = 1, bachelor’s degree or above = 2); marital status (single/separated/widowed/divorced = 1, married = 2) and residence area (1 = Hong Kong Island, 2 = Kowloon, 3 = New Territory) were collected. 

Little consistency could be found in the measurements of HIV knowledge used in the existing literature [25]. Based on our research purpose, the Chinese Questionnaire on HIV Prevention and Awareness, developed by Ministry of Education (Taiwan), was adopted to measure individuals’ knowledge, attitudes, and awareness. For the knowledge subscale, 55 items were included covering the prevalence (one item), definition (four items), transmission routes (seven items), misunderstanding (26 items), risk behaviors (six items), as well as symptoms and treatment (11 items) of HIV. The Cronbach’s alpha was 0.87, suggesting a good reliability. 

For each item, participants were asked to respond with “true”, “false”, or “I don’t know”. A total score of knowledge was generated by counting the number of correct responses from all the items. A higher score indicated greater knowledge about HIV.

HIV attitude and awareness. The attitude subscale included 34 items in total, assessing individual’s attitude toward the treatment and prevention of HIV. It included the acceptance of people living with HIV, shame and guilt, risk perception, and awareness of prevention and disease control. A five-point Likert scale was used to assess individual’s attitude, ranging from “1” = “strongly disagree” to “5” = “strongly agree”. The scale showed a Cronbach’s alpha of 0.828. 

Given no psychometric properties of the questionnaires were reported, we have conducted an exploratory factor analysis (EFA) by using a principal–component factor extraction. Based on the original structure of the instrument, a three-factor solution was tested. The KMO value (0.906) suggested superb sampling adequacy. Given that the attitude questionnaire was conceived as a multidimensional construct with dimensions being nonorthogonal, an oblique rotation was used. With such a rotation, three factors were generated with sums of squared loadings ranging from 4.29 to 7.92. The first factor accounted for 28.69% of the variance, with the second accounting for 10.97%, and the third 10.09%. In total, 49.74% of variance was explained by the three factors. The clustering of items into different factors was quite interpretable, which could be labeled as acceptance, stigma (i.e., shame, guilt, and risk perception) and awareness. The first factor “acceptance”, referring to the attitude of accepting and including people with HIV in one’s social network, had 19 items (e.g., accepting the AIDS patients can encourage them to face their lives bravely). A Cronbach’s alpha of 0.93 was obtained for the acceptance subscale. The second factor “stigma”, including seven items, referred to the negative emotion and attitude toward the disease and affected population (e.g., I think AIDS is a punishment from God). It obtained a Cronbach’s alpha of 0.65. The third factor, “awareness”, was characterized by the perceptions about the epidemic influences of HIV, related public policy, and resource allocation to help PLWH (e.g., I think that the government should provide more resources to HIV infected people). Eight Items were included, and a good reliability was indicated (Cronbach’s alpha = 0.81). 

### 2.3. Statistical Analysis

Using the approach of Baron & Kenny [26], a mediation model was conducted to analyze the direct and indirect effect of HIV knowledge on awareness. Specifically, the association between knowledge and awareness was the total effect (path C), which consisted of a direct effect of knowledge (path C*) and two indirect effects through acceptance and stigma. The direct path (path C*) refers to the relationship between knowledge and awareness, while the two indirect paths captured the effect of knowledge via affecting stigma and acceptance. The associations between knowledge and acceptance (path A1) and stigma (path A2) were path A, and the associations from acceptance (path B1) and stigma (path B2) to awareness were path B. The significance of mediating effects was assessed on the basis of the bias-correction bootstrapped 95% confidence intervals, using PROCESS Macro 3.0, SPSS Statistics 24 [27]. All the analysis was adjusted for participants’ demographic variables including age, sex, education and marital status. 

## 3. Results

### 3.1. Descriptive Results

Eight hundred and ten participants aged from 18 to 33 years (Mean = 20.63 ± 2.05) were randomly recruited to take part in the study, and 804 completed the questionnaire. 41.7% of the participants were male, 57.5% were female, with seven participants (0.9%) identifying themselves as transgender. Given that most participants had not graduated at the time of data collection, the educational attainment was diploma or associate degree (68.6%), with 31.4% having an educational level higher than bachelor’s degree. The majority of the participants (97.4%) were not married, most likely single (96.8%). The participants were recruited from universities in different districts in Hong Kong, with 58.1% from the New Territories, 29.1% from Kowloon, and 12.8% from Hong Kong Island. 

The average rate of correct responses to the HIV knowledge questions was 65.74% across the 55 items on HIV among Hong Kong young people. The results of t-test showed the only significant difference of knowledge was found when comparing high and low education groups, that people with higher education showed better HIV knowledge (Mean = 37.23 ± 7.37) compared with those with lower education (M = 35.68 ± 8.75), *p* = 0.011. It was noteworthy that approximately 50% of the participants believed that HIV could be transmitted via mosquito bite (48.1%) or sharing a toothbrush (53.6%), and over 60% failed to identify anal sex as the primary route of HIV infection. The results indicated that the misunderstanding about HIV transmission is still widespread among the young generation.

Most participants showed an accepting attitude toward people with HIV. Sixteen items of acceptance received “agree” or “strongly agree” from over 60% of the participants, such as “I am willing to accept people with HIV and be their friend” and “I am willing to do sports/shake hands with people with HIV”. The item that the least number of people (48.8%) agreed with was “I am willing to let the organizations that serve AIDS patients be built near my home”, suggesting that concern and worry is pervasive among the lay public. For the statements about stigma, the rates of “agree” or “strongly agree” responses were much lower (29.7%). The lowest rate of “agree” was found on “I think that taking HIV test is disgraceful” (11.2%) and “I think AIDS is a punishment from God” (15.4%). The results suggested a relatively low level of discrimination and isolation intention in the young generation. Meanwhile, 79.8% of the participants chose “agree” or “strongly agree” with the statements on the awareness about the risk of HIV infection, its social impact, and the importance of prevention and control.

### 3.2. Predicting HIV Awareness

The results of correlation analysis are displayed in Table 1. 

With a linear regression model adjusting for the demographic variables (i.e., gender, age and education), knowledge was found to have a positive effect on awareness (B = 0.023, *p* < 0.001), confirming the significance of path C. When conducting the mediating analysis to test the direct effect of knowledge and indirect effects through stigma and acceptance (see Table 2), knowledge was also found to be associated with higher acceptance (B = 0.023, *p* < 0.001), but not with stigma (B = −0.001, *p* = 0.58), which confirmed one part of path A.

**Figure 1:**
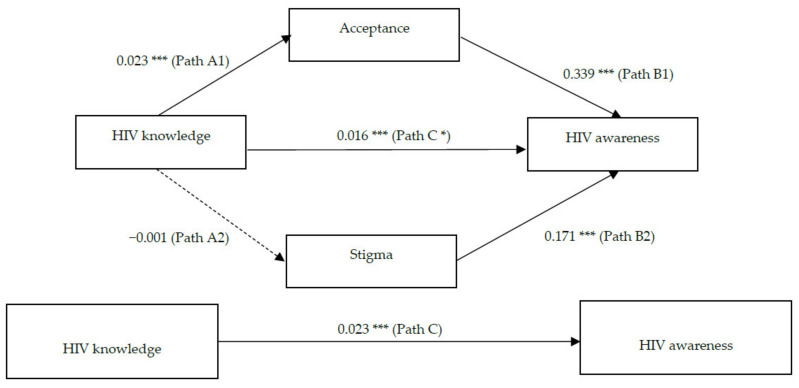
The mediation pathways of HIV-related knowledge, attitude (acceptance and stigma) and awareness. * *p* ≤ 0.05; ** *p* ≤ 0.01; *** *p* ≤ 0.001.

Meanwhile, the mediation analysis showed that acceptance was related with higher levels of awareness (B = 0.339, *p* < 0.001), and interestingly, stigma was also related to increased awareness (B = 0.171, *p* < 0.001). The results have confirmed path B between mediators and awareness. With bootstrapping analysis to test the mediation effects, a significant total indirect effect was found (B = 0.008, 95% CI = [0.005, 0.011]). However, only acceptance yielded a significant indirect effect (B = 0.008, 95%CI = [0.006, 0.011]), not stigma (B = −0.000, 95%CI = [−0.001, 0.001]). In addition, the direct effect of knowledge was still significant after the mediators entering the model, B = 0.016, *p* < 0.001, 95%CI = [0.011, 0.020]. The results confirmed a partial mediating role of acceptance on the positive effect of HIV knowledge in awareness (see Figure 1).

## 4. Discussion

Using a sample of 810 college students in Hong Kong, our study investigated the levels of knowledge about, attitudes toward, and awareness of HIV among younger adults. By addressing the roles of knowledge and attitudes in predicting awareness, the findings provide an updated and comprehensive profile of HIV-related concepts among the Hong Kong youths.

As shown in the results, the overall rate of correct responses to the questions on HIV-related knowledge was 65.74%, suggesting a moderate level among the college students. However, it is noteworthy that misconceptions on the HIV transmission route still exist widely in the young generation, for example, about 60% failed to recognize that anal sex is the primary sexual contact for HIV transmission. A survey among Hong Kong adolescents conducted twenty years ago reported that over 20% of the participants believed that HIV can be transmitted by mosquitoes or sharing a cigarette [6]. In comparison, our results (i.e., 50% believed that HIV could be passed via insects or sharing toothbrush) suggested that the knowledge and understanding among the young generation about HIV transmission showed no significant improvements in the past two decades, if not worsened. It is possible that the discrepancies in the findings stem from different measures for HIV knowledge. A recent study suggested that the overall level of knowledge regarding HIV transmission among Hong Kong youth was relatively good (mean score of correct responses was 3.55 out of 4 [11]. However, only one item on HIV transmission routes was included in this study (i.e., one can get AIDS by sharing drug needles), making it difficult to capture a bigger picture of young generation’s situation. Taken together, our findings indicate that lack of knowledge and the misconceptions about HIV could still be the major reasons for people’s low awareness, thus forming obstacles for the prevention and control of HIV infection. Therefore, educational programs to provide information, particularly focusing on the transmission routes of HIV, should be developed for college students.

Meanwhile, our results on attitudes showed a more optimistic picture, such that most participants reported an accepting, instead of stigmatizing, attitude toward people with HIV. In addition, acceptance of PLWHs was found to be a partial mediator in the positive effects of knowledge on HIV awareness. Previous studies have found that limited knowledge about HIV was a major contributing factor in the low acceptance of HIV counselling and testing [28], and our results showed that it is also a predictor for lower levels of person-centered attitudes, in terms of willingness to accept and help PLWHs. In addition, a knowledge-related increase in acceptance was associated with higher levels of awareness and support. The results clearly support that accurate information about HIV’s transmission, prevention and treatment, as well as how to conduct safe sex, would raise people’s acceptance and compassion toward the HIV infected population. Therefore, the findings indicate that intervention programs aiming to prevent and control the spread of HIV among college students in Hong Kong should focus on knowledge-based programs, such as organizing education workshops and knowledge sharing sessions on college campus.

It is noteworthy that no mediating effect was found on stigma, although stigma was associated with a higher level of awareness. A possible explanation is that stigma could put people on alert, resulting in greater perceptions about the importance of disease control. Therefore, a positive association was found between stigma and awareness. Unfortunately, with the factors included in the current study, further exploration to understanding this relationship is not possible. Another limitation of the present study is that we failed to identify the potential moderators. For example, an individual’s own sexual orientation may influence the attitude and awareness of HIV or PLWH, thus sexual orientation should be included in future studies. In addition, the causal relationship between knowledge and awareness was not clear in the current study. It is possible that people with higher levels of awareness about HIV are more likely to seek out and take in relevant information or knowledge. Therefore, future studies using experimental manipulation might be necessary to better understand this relationship. Finally, more longitudinal evidence is needed to delineate the trajectory of how knowledge of, and attitudes toward, HIV have changed across time and how they influence people’s sex behaviors during emergent adulthood.

## 5. Conclusions

The findings suggest that both knowledge and attitude are key determinants in people’s awareness of this disease. Greater knowledge exerts a direct effect in predicting higher levels of awareness; meanwhile, it also has an indirect effect through promoting people’s accepting attitudes toward those living with HIV. The findings have significant insights for developing educational and intervention programs for the young generations in Hong Kong. Sex education among college students should pay more attention to minimizing the HIV knowledge gaps, and increasing risk perceptions, as well as responsibility of self-protection in sex [29]. For instance, knowledge-sharing workshops, e.g., on safe sex and HIV transmission routes, should be provided at campuses, aiming to eliminate college students’ misconceptions, and increase their risk perception. Meanwhile, the mediation pathway of attitude in the effect of knowledge was addressed, which suggests the importance of promoting person-centered attitudes in the programs advocating awareness at college campus. For example, programs such as human library could be held to invite people living with HIV to share their experiences with college students. A positive relationship between stigma and awareness indicates the possibility that stigma may motivate people to know more about HIV prevention and control, thus increase the public awareness about this disease. 

## Figures and Tables

**Table 1:** Correlations between demographic variables, knowledge, acceptance, stigma and awareness.

	Education	Gender	HIV Knowledge	Acceptance	Stigma	Awareness
Age	0.172 **	−0.168 **	0.039	−0.040	0.011	−0.117 **
Gender	−0.035	1				
HIV knowledge	0.086 *	0.016	1			
Acceptance	0.068	0.035	0.353 **	1		
Stigma	0.045	−0.059	−0.023	−0.304 **	1	
Awareness	0.016	0.074 *	0.342 **	0.371 **	0.056	1

* *p* ≤ 0.05; ** *p* ≤ 0.01.

**Table 2:** Regression analyses examining the mediating effects of acceptance and stigma on the association between knowledge and awareness.

Baron and Kenny’s Four-Step Approach	B (95% CI)	*p*-Value
Step 1 (Path C)		
Association between HIV knowledge and awareness		
(DV: HIV awareness ^#^; IV: knowledge)	0.023 (0.0186, 0.0279)	<0.001
Step 2 (Path A)		
Association between knowledge and mediators		
(DV: Acceptance; IV: knowledge about HIV)	0.023 (0.019, 0.028)	<0.001
(DV: Stigma; IV knowledge about HIV/AIDS)	−0.001 (−0.006, 0.004)	0.584
Step 3 (Path B)		
Associations between the mediators and awareness ^#^ with adjustment for knowledge		
(DV: awareness ^#^; IV: acceptance)	0.339 (0.263, 0.415)	<0.001
(DV: awareness ^#^; IV: stigma)	0.171 (0.104, 0.238)	<0.001
Step 4 (Path C *)		
Association between knowledge and awareness ^#^ with adjustment for acceptance and stigma		
(DV: awareness ^#^; IVs: knowledge, acceptance and stigma)		
Knowledge	0.016 (0.011, 0.020)	<0.001
Indirect effects		
Total indirect effect of knowledge on awareness ^#^ through acceptance and stigma	0.008 (0.005, 0.011)	
Indirect effect of knowledge on awareness ^#^ through acceptance	0.008 (0.006, 0.011)	
Indirect effect of knowledge on awareness ^#^ through stigma	−0.000 (−0.001, 0.001)	

^#^ Awareness level was adjusted for age, sex and education. DV: dependent variable of the underlying regression model; IV: independent variable of the underlying regression model; B: regression coefficient; CI: confidence interval.

## Data Availability

Data, analytic methods, and study materials can be made available upon request from the corresponding author. The study was not preregistered.
